# Effectiveness and safety of Breviscapine in the treatment of coronary heart disease: a meta-analysis of randomized controlled trials

**DOI:** 10.3389/fcvm.2026.1770569

**Published:** 2026-09-11

**Authors:** Haoyang Yu, Chunyu Wen, Zhigang Yang, Gen Li, Jienan Luan, Hua Zhou, Yuanyuan Li, Mingtai Chen, Mengnan Liu

**Affiliations:** 1 Shenzhen Traditional Chinese Medicine Hospital, Guangzhou University of Chinese Medicine, Shenzhen, Guangdong, China; 2 Chinese Medicine Guangdong Laboratory (Hengqin Laboratory), Guangdong-Macao In-Depth Cooperation Zone in Hengqin, Zhuhai, China; 3 Traditional Chinese Medicine Syndrome, Guangdong Provincial Hospital of Chinese Medicine, Guangdong Provincial Academy of Chinese Medical Sciences, The Second Affiliated Hospital of Guangzhou University of Chinese Medicine, Guangzhou, China; 4 Faculty of Chinese Medicine and State Key Laboratory of Mechanism and Quality of Chinese Medicine, Macau University of Science and Technology, Taipa, Macao SAR, China; 5 Department of Cardiovascular Medicine, the Affiliated Hospital of Traditional Chinese Medicine, Southwest Medical University, Luzhou, Sichuan, China

**Keywords:** Breviscapine, coronary heart disease, meta-analysis, protocol, randomized trial

## Abstract

**Background:**

Coronary heart disease is a leading cause of cardiovascular mortality. Residual angina pectoris often persists despite long-term treatment. Breviscapine, a plant-derived flavonoid, exhibits multi-target cardiovascular effects; however, its clinical role requires systematic evaluation.

**Objective:**

This meta-analysis aims to comprehensively assess the efficacy and safety of Breviscapine for angina pectoris in coronary heart disease.

**Methods:**

Databases were systematically searched for relevant randomized controlled trials (RCTs) up to December 2024. Two researchers independently performed study selection, data extraction, and risk-of-bias assessment. Data were analyzed using RevMan 5.4, employing a random-effects model for all meta-analyses.

**Results:**

A total of 29 RCTs were included. Breviscapine (alone or add-on) demonstrated significant benefits over conventional therapy in: (1) Total effective rate (13 studies, RR = 1.20, 95% CI 1.14–1.26, *p* < 0.00001). (2) Angina symptom improvement rate (11 studies, RR = 1.23, 95% CI 1.16–1.30, *p* < 0.00001). (3) Electrocardiogram improvement rate (13 studies, RR = 1.23, 95% CI 1.16–1.30, *p* < 0.00001). (4) TCM syndrome score (2 studies, RR = 1.32, 95% CI 1.07–1.61, *p* = 0.008). In addition, Breviscapine showed exploratory trends toward improvement in lipid profiles, with reductions in TG, TC, and LDL-C reported across the included studies, although high heterogeneity and the limited number of studies precluded quantitative pooling. For HDL-C, only two studies reported this outcome, with one showing an increase and the other showing no significant change. The incidence of adverse reaction did not differ significantly between groups (5 studies, *p* = 0.39). Data on acute cardiovascular events and all-cause mortality were insufficient or unreported.

**Conclusion:**

Breviscapine, alone or as add-on therapy, significantly improves angina symptoms and electrocardiographic findings, with exploratory trends toward favorable lipid profile changes observed in the included studies, indicating promising efficacy and short-term clinical tolerability. However, the evidence is limited by methodological concerns in the included studies and substantial heterogeneity in lipid outcomes. The safety of Breviscapine requires further investigation. Higher-quality RCTs are warranted to strengthen these conclusions.

**Systematic Review Registration:**

https://www.crd.york.ac.uk/PROSPERO/view/CRD420251055996, PROSPERO CRD420251055996.

## 1 Introduction

Cardiovascular diseases (CVDs), particularly coronary heart disease (CHD) and stroke, represent the predominant cause of global mortality and a significant contributor to disability (1). CHD continues to exhibit high morbidity and mortality rates, imposing a substantial economic burden on societies (2, 3). Its pathological progression is influenced by multiple factors, including inflammatory responses and disruptions in lipid, amino acid, carbohydrate, and bile metabolism (4–6). In its early stages, CHD manifests as coronary atherosclerosis, characterized by lipid deposition and chronic inflammation. Beyond increasing mortality risk, CHD significantly impairs patients' daily functioning and quality of life (QOL) (7, 8). Although conventional treatments such as revascularization and pharmacological interventions are widely employed, many CHD patients still experience persistent angina-related symptoms and adverse effects, including dizziness and headache (9–12). Given these limitations, herbal medicine—with its millennia of therapeutic application—may offer a viable complementary approach to CHD management.

Breviscapine, a bioactive flavonoid derived from *Erigeron breviscapu*s (13), exhibits multiple pharmacological properties, including microvascular dilation, reduction in blood viscosity, and improvement in microcirculation, as demonstrated in both experimental and clinical studies (14). This compound has demonstrated protective effects against ischemia-reperfusion injury in various organs, particularly the heart and brain (15). Preclinical research using animal models of cardiovascular disease has revealed its anti-inflammatory effects on myocardial tissue (16), with further evidence suggesting its capacity to mitigate inflammatory responses and consequently reduce myocardial ischemia-reperfusion injury (MIRI) in diabetic rats (17). Clinical investigations have increasingly documented the therapeutic potential of Breviscapine in the management of coronary heart disease. As a natural extract with blood-activating and stasis-resolving properties, it demonstrates coronary artery dilation, microcirculatory enhancement, plaque stabilization, and improvement in myocardial ischemia. Growing clinical evidence supports its efficacy in alleviating angina symptoms and enhancing quality of life in CHD patients (18–20).

Despite the growing number of clinical studies and reviews examining Breviscapine's application in CHD patients, the current evidence remains limited by methodological weaknesses. Notably, no comprehensive meta-analysis has systematically evaluated both the efficacy and safety of Breviscapine for CHD treatment. To address this critical evidence gap, we conducted a systematic meta-analysis to evaluate Breviscapine as an evidence-based add-on therapy and to provide robust clinical evidence regarding its therapeutic value.

## 2 Methods and analysis

### 2.1 Registration

This protocol has been registered on the International Prospective Register of Systematic Review (PROSPERO). The trial registration number of PROSPERO is CRD420251055996. The procedure of this protocol was conducted according to the Preferred Reporting Items for Systematic Review and Meta-analysis Protocols (PRISMA 2020) guidance.

### 2.2 Inclusion criteria

Only trials meeting the following three criteria were included:

1. Type of study

We included all randomized controlled trials (RCTs) investigating the effectiveness and safety of Breviscapine combined with conventional medication for the treatment of CHD, published in either Chinese or English.

2. Participants

The study included patients diagnosed with CHD regardless of age, sex, ethnicity, education, or economic status, and irrespective of whether they were outpatients or inpatients. The diagnostic criteria for CHD were confirmed according to one of the past or current definitions, including the “Nomenclature and criteria for diagnosis of ischemic heart disease” (21) or the “ACC/AHA 2002 guideline update for the management of patients with chronic stable angina” (22).

3. Interventions

Breviscapine in tablet, injection, or any other dosage form, administered alone or combined with the control group treatment, regardless of dose or treatment duration. If Breviscapine in the intervention group is combined with other Traditional Chinese Medicine preparations, the study was excluded excluded.

4. Outcome

The primary outcome measures included: mortality; acute cardiovascular events; total efficacy rate; improvement of angina symptoms; and adverse reactions. The secondary outcome measures included: changes in electrocardiogram; levels of total cholesterol (TC), triglyceride (TG), low-density lipoprotein cholesterol (LDL-C), and high-density lipoprotein cholesterol (HDL-C); and TCM syndrome score.

### 2.3 Exclusion criteria

Studies meeting any of the following criteria will be excluded:

1. Non-randomized controlled trials (including observational cohort studies, case-control studies, case reports, experimental studies, and reviews).
2. Study populations comprising CHD patients complicated with severe respiratory diseases, acute infectious diseases, severe cardiac diseases, severe hepatic diseases, tumors, or severe anxiety/depression.
3. Interventions that do not meet the inclusion criteria.
4. Studies that do not employ appropriate outcome measures for evaluation.
5. Studies in which the experimental group has an abnormal treatment duration or dosage.

### 2.4 Search strategy

The following electronic bibliographic databases will be searched from inception to December 2024: PubMed, Embase, the Cochrane Library, China National Knowledge Infrastructure (CNKI), Chinese Scientific Journals Database (VIP), and Wanfang Database. No language restrictions will be applied. The search will be limited to clinical trials. The following sources will also be searched to identify ongoing or completed clinical trials: ClinicalTrials.gov and the World Health Organization (WHO) International Clinical Trials Registry Platform. Additional relevant studies will be retrieved from the reference lists of systematic reviews and included studies. Controlled vocabulary will be used where possible. In addition, the search strategy for title, abstract, and keyword fields will be adapted according to the characteristics of each database. Search terms are grouped into three blocks (see Table 1).

**Table 1:** Search items of effectiveness and safety of breviscapine in the treatment of coronary heart disease.

Search block	Search items
Participants	Angina Pectoris OR Anginas, Stable OR Stable Angina OR Stable Anginas OR Chronic Stable Angina OR Angina, Chronic Stable OR Anginas, Chronic Stable OR Chronic Stable Anginas OR Stable Angina, Chronic OR Stable Anginas, Chronic OR Angina Pectoris, Stable OR Angina Pectori, Stable OR Pectori, Stable Angina OR Pectoris, Stable Angina OR Stable Angina Pectori OR Stable Angina Pectoris OR Stenocardia OR Stenocardias OR Angor Pectoris OR Coronary Diseases OR Disease, Coronary OR Diseases, Coronary OR Coronary Heart Disease OR Coronary Heart Diseases OR Disease, Coronary Heart OR Diseases, Coronary Heart OR Heart Disease, Coronary OR Heart Diseases, Coronary OR Anginas, Unstable OR Unstable Anginas OR Angina at Rest OR Angina, Preinfarction OR Anginas, Preinfarction OR Preinfarction Angina OR Preinfarction Anginas OR Unstable Angina OR Angina Pectoris, Unstable OR Angina Pectori, Unstable OR Unstable Angina Pectori OR Unstable Angina Pectoris OR Myocardial Preinfarction Syndrome OR Myocardial OR Preinfarction Syndromes OR Preinfarction Syndrome, Myocardial OR Preinfarction Syndromes, Myocardial OR Syndrome, Myocardial Preinfarction OR Syndromes, Myocardial Preinfarction
Intervention	Breviscapine OR Drugs, Chinese Herbal OR Chinese Drugs, Plant OR Chinese Herbal Drugs OR Herbal Drugs, Chinese OR Plant Extracts, Chinese OR Chinese Plant Extracts OR Extracts, Chinese Plant OR Medicine, Chinese Traditional OR Traditional Chinese Medicine OR Chung I Hsueh OR Hsueh, Chung I OR Traditional Medicine, Chinese OR Zhong Yi Xue OR Chinese Traditional Medicine OR Chinese Medicine, Traditional
Study design	Randomized controlled trial OR controlled clinical trial OR randomized OR placebo OR drug therapy OR randomly OR trial OR groups

### 2.5 Study selection and data extraction

**Figure 1:**
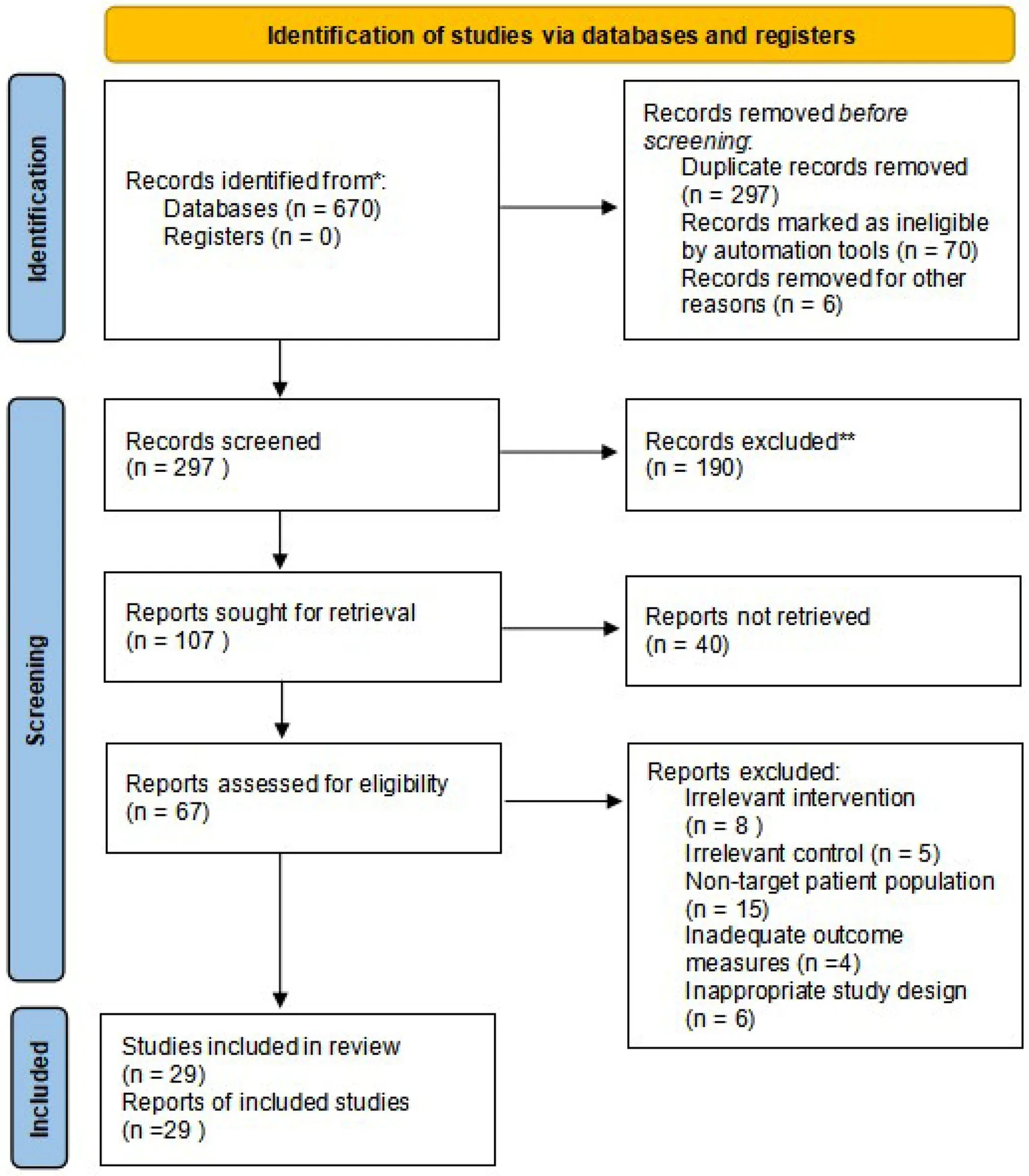
PRISMA flow diagram, literature search and review flowchart for selection of studies.

Literature retrieved from the search were managed using EndNote X9 software. Two authors independently screened the titles and abstracts of all studies retrieved from the above electronic databases to identify potentially eligible studies. Articles that are duplicates or do not meet the eligibility criteria, intervention, or outcome requirements of this study were excluded. After finalizing the eligible articles, data from the included studies were independently extracted by two authors. Disagreements were resolved through discussion or by arbitration from a third party if necessary. The following data items were extracted: first author, publication year, diagnosis information, age, sex, trial characteristics, interventions and controls, participants, study methodology, outcomes, adverse events, etc (see Figure 1).

### 2.6 Risk of bias assessment

The methodological quality of the eligible studies was evaluated using the Cochrane Risk of Bias 2.0 (RoB 2) tool. The assessment includes the following domains: randomization process (D1), deviations from intended interventions (D2), missing outcome data (D3), measurement of the outcome (D4), and selection of the reported result (D5). Each domain were assessed as “low risk,” “high risk,” or “some concerns” according to the detailed descriptions of the eligible studies.

### 2.7 Data synthesis and statistical analysis

Meta-analyses were performed using a Mantel-Haenszel (M-H) random-effects model with DerSimonian-Laird estimation for between-study variance (*τ*^2^), as implemented in RevMan 5.4. A random-effects model was applied for all meta-analyses because clinical and methodological heterogeneity across studies was anticipated (e.g., variations in patient populations, Breviscapine dosage and formulation, treatment duration, and control interventions). This approach was applied irrespective of the observed *I*^2^ value, as it provides more conservative and generalizable estimates than a fixed-effect model when heterogeneity is expected. Heterogeneity was quantified using the *I*^2^ statistic, with values of 25%, 50%, and 75% considered as low, moderate, and high heterogeneity, respectively. Tau^2^ was reported alongside *I*^2^ to describe the magnitude of between-study variance. All analyses were conducted using RevMan 5.4 (23).

### 2.8 Sensitivity analysis, subgroup analysis, and meta-regression

Sensitivity analyses and subgroup analyses were planned to explore potential sources of heterogeneity when substantial heterogeneity was detected. However, due to the limited number of studies available for meaningful subgroup exploration, such analyses were not performed for most outcomes. Meta-regression was not conducted because of the insufficient number of studies to support reliable regression modeling. If data extraction was insufficient for quantitative synthesis, a qualitative synthesis was conducted instead.

### 2.9 Publication bias

A funnel plot was generated to evaluate reporting bias among the included studies.

### 2.10 Quality of evidence

The quality of evidence for the main outcomes was assessed using the Grading of Recommendations Assessment, Development and Evaluation (GRADE) approach. Five domains were evaluated, including limitations in study design, inconsistency, imprecision, indirectness, and publication bias.

### 2.11 Patient and public involvement

Patients and/or the public were not involved, as this study is based on secondary source analysis.

## 3 Results

### 3.1 Study identification

The process of study selection and identification is illustrated in Figure 1. Initially, a total of 670 potentially relevant records were screened in electronic databases. After removing duplicates (*n* = 297), records marked as ineligible by automation tools (*n* = 70), and those excluded for other reasons (*n* = 6), 297 records were selected for further screening. Following title and abstract screening, 190 records were excluded because they were not randomized controlled trials. After removing reports that were not retrieved (*n* = 40), 67 full-text reports were assessed for eligibility, of which 38 were excluded for the following reasons: non-target patient population (*n* = 15), irrelevant intervention or exposure (*n* = 8), irrelevant control (*n* = 5), inappropriate study design (*n* = 6), and inadequate outcome measures (*n* = 4). Ultimately, 29 full-text studies were included.

Su YC 2015 was retained in this meta-analysis because Salvia miltiorrhiza injection was used equally in both the treatment and control groups as part of conventional background therapy; the only difference between groups was the addition of Breviscapine in the treatment group. The use of Salvia miltiorrhiza injection did not introduce between-group imbalance. Furthermore, a sensitivity analysis excluding this study confirmed that its inclusion did not materially alter the overall effect estimates for any outcome (total efficacy rate: RR 1.21 95% CI 1.15–1.27, *p* < 0.00001, *I*^2^ = 0%; ECG improvement: RR 1.23 95% CI 1.16–1.31, *p* < 0.00001, *I*^2^ = 0%; adverse reactions: RR 0.95 95% CI 0.43–2.11, *p* = 0.90, *I*^2^ = 0%), confirming that the inclusion of this study did not drive the overall results.

### 3.2 Characteristics of the included studies

The basic details of the 29 included studies (24–52) are shown in Table 2. Thirteen studies (24, 27, 28, 30–34, 37, 38, 41, 49, 52) reported total efficacy rate, of which 2 studies (30, 37) simultaneously reported changes in electrocardiogram (ECG) readings. 11 studies reported improvement in angina symptoms, of which 6 studies (39, 44–46, 51) simultaneously reported ECG efficacy and 1 study (48) reported blood lipid levels. Thirteen studies reported changes in ECG readings, of which 1 studies (30) reported blood lipid levels while another study (32) also reported blood lipid levels. Two studies (36, 44) reported changes in TCM symptom scores. Five studies (30, 32, 34, 36, 47) reported adverse reaction.

**Table 2:** Characteristics of included studies.

Study ID	Age	Study design	Group	Sample size	Withdrawal size	Dosage and usage	Duration (*t*/*d*)	Outcome measure	Mortality reported
Yang (24)	T:60.24 ± 7.02	RCT	TG	55	0	CWMT + Breviscapine 50 mg qd	14	A	NR
	C:59.74 ± 6.28		CG	55	0	CWMT	14		NR
Zhao et al. (25)	–	RCT	TG	180	0	Breviscapine 176 mg tid	28	C	NR
			CG	60	0	CWMT	28		NR
Gao (26)	T:59.01 ± 6.57	RCT	TG	48	0	CWMT + Breviscapine 50 mg qd	14	C	NR
	C:58.94 ± 6.27		CG	48	0	CWMT	14		NR
Yang et al. (28)	62.46 ± 1.32	RCT	TG	43	0	CWMT + Breviscapine 20 mg qd	14	A, C	NR
			CG	43	0	CWMT	14		NR
Jiang et al. (27)	T:65.64 ± 3.51	RCT	TG	30	0	CWMT + Breviscapine 50 mg qd	14	A	NR
	C:65.14 ± 3.67		CG	30	0	CWMT	14		NR
Qiang (29)	48–73 years	RCT	TG	30	0	Breviscapine 50 mg qd	14	B	NR
			CG	30	0	CWMT	14		NR
Su (30)	T:63.50 ± 1.50	RCT	TG	51	0	CWMT + SM 200mg + Breviscapine 20 mL qd	14	A, C, D, F	NR
	C:62.00 ± 1.50		CG	51	0	CWMT + SM 200mg	14		NR
Li and Wang (32)	T:45–69 years	RCT	TG	65	0	Breviscapine 20–40 mL qd	28	A, D, F	NR
	C:40–70 years		CG	65	0	CWMT	28		NR
Din (31)	55–66 years	RCT	TG	53	0	CWMT + Breviscapine 40 mg tid	60	A	NR
			CG	52	0	CWMT	60		NR
Wang et al. (33)	T:64.70 ± 1.40	RCT	TG	36	0	CWMT + Breviscapine 50 mg qd	14	B, C	NR
	C:64.80 ± 1.60		CG	36	0	CWMT	14		NR
Liang (35)	T:54.80 ± 7.90	RCT	TG	32	0	CWMT + Breviscapine 25 mg qd	15	B, C	NR
	C:53.30 ± 8.60		CG	32	0	CWMT	15		NR
Duan (34)	T:61.32 ± 3.90	RCT	TG	62	0	CWMT + Breviscapine 80 mg qd	10	A, F	NR
	C:53.30 ± 8.60		CG	58	0	CWMT	10		NR
Shen et al. (36)	T:69.89 ± 9.32	RCT	TG	53	0	CWMT + Breviscapine 20 mL qd	14	B, C, E, F	NR
	C:68.04 ± 9.81		CG	51	0	CWMT	14		NR
Huang (37)	40–86 years	RCT	TG	61	0	CWMT + Breviscapine 50 mg qd	14	A, C	NR
			CG	61	0	CWMT	14		NR
Dai (38)	T:73.50 ± 2.50	RCT	TG	60	0	CWMT + Breviscapine 50 mg qd	Not mentioned	A	NR
	C:72.30 ± 2.10		CG	60	0	CWMT	Not mentioned		NR
Wang et al. (39)	T:58.00 ± 9.10	RCT	TG	45	0	CWMT + Breviscapine 40 mL qd	14	B, C	NR
	C:60.00 ± 8.50		CG	45	0	CWMT	14		NR
Yang et al. (40)	T:62–82 years	RCT	TG	64	0	CWMT + Breviscapine 50 mg qd	14	B	NR
	C:62–83 years		CG	60	0	CWMT	14		NR
Meng and Yu (42)	T:56.80 ± 9.10	RCT	TG	41	0	CWMT + Breviscapine 40 mg qd	14	B	NR
	C:58.30 ± 9.90		CG	39	0	CWMT	14		NR
Tan (43)	T:65.00 ± 6.21	RCT	TG	30	0	CWMT + Breviscapine 40 mg qd	14	B	NR
	C:66.00 ± 7.28		CG	30	0	CWMT	14		NR
Qin (41)	63. 6 ± 7. 9	RCT	TG	39	0	CWMT + Breviscapine 50 mg qd	14	A	NR
			CG	39	0	CWMT	14		NR
Shi (45)	35–70 years	RCT	TG	42	0	CWMT + Breviscapine 50 mg qd	14	B, C	NR
			CG	40	0	CWMT	14		NR
Liang and Suwen (44)	T:42–78 years	RCT	TG	110	0	CWMT + Breviscapine 30 mg qd	15	B, E	NR
	C:40–77 years		CG	110	0	CWMT	15		NR
Li (47)	T:70.00 ± 5.20	RCT	TG	51	0	CWMT + Breviscapine 40 mg qd	30	C, F	NR
	C:73.00 ± 8.60		CG	51	0	CWMT	30		NR
Hu and Tao (46)	T:57.7	RCT	TG	50	0	CWMT + Breviscapine 50 mg qd	14	B, C	NR
	C:60.9		CG	50	0	CWMT	14		NR
Peng and Ye (48)	T:60.00 ± 5.77	RCT	TG	30	0	CWMT + Breviscapine 50 mg qd	14	B, D	NR
	C:58.00 ± 7.50		CG	32	0	CWMT	14		NR
Zhou and Yousheng (49)	T:59.52 ± 6.35	RCT	TG	35	0	CWMT + Breviscapine 50 mg qd	14	A	NR
	C:57.55 ± 8.40		CG	35	0	CWMT	14		NR
Zhao et al. (51)	T:68.7	RCT	TG	30	0	CWMT + Breviscapine 100 mg qd	14	B, C	NR
	C:66.1		CG	30	0	CWMT	14		NR
Shi (50)	T:62.30 ± 7.1	RCT	TG	80	0	CWMT + Breviscapine 30 mg qd	14	C	NR
	C:61.40 ± 9.1		CG	46	0	CWMT	14		NR
Qu (52)	T:43–74 years	RCT	TG	36	0	CWMT + Breviscapine 20 mL qd	21	A	NR
	C:40–72 years		CG	32	0	CWMT	21		NR

NR indicates that the study did not report mortality data. A, total efficacy rate; B, improvement of angina symptoms; C, changes of electrocardiogram; D, levels of blood lipid; E, TCM syndrome score; F, adverse reaction; CWMT, conventional western medicine treatment; TG, treatment group; CG, control group; SM, salvia miltiorrhiza injection.

The criteria used to judge treatment success varied across studies. Four studies (32, 39, 42, 48) referred to the 2002 “Guiding Principles for Clinical Research of New Chinese Medicines.”10 studies (24, 29, 36–38, 40, 41, 45, 50, 51) listed efficacy criteria but did not cite a specific source. 2 studies (31, 47) referred to the 1993 “Guiding Principles for Clinical Research of Cardiovascular Drugs.” One study referred to the 1987 version of those principles. Two studies (25, 43) did not list or reference any efficacy criteria. Seven studies (26, 27, 30, 33–35, 37) referred to efficacy criteria from other published literature. One study (52) used criteria revised at a national symposium in 1979, 1 study (49) referenced an unspecified version of the cardiovascular drug research guidelines, 1 study (28) used criteria from a medical reference book.

### 3.3 Evaluation of methodological quality

**Figure 2:**
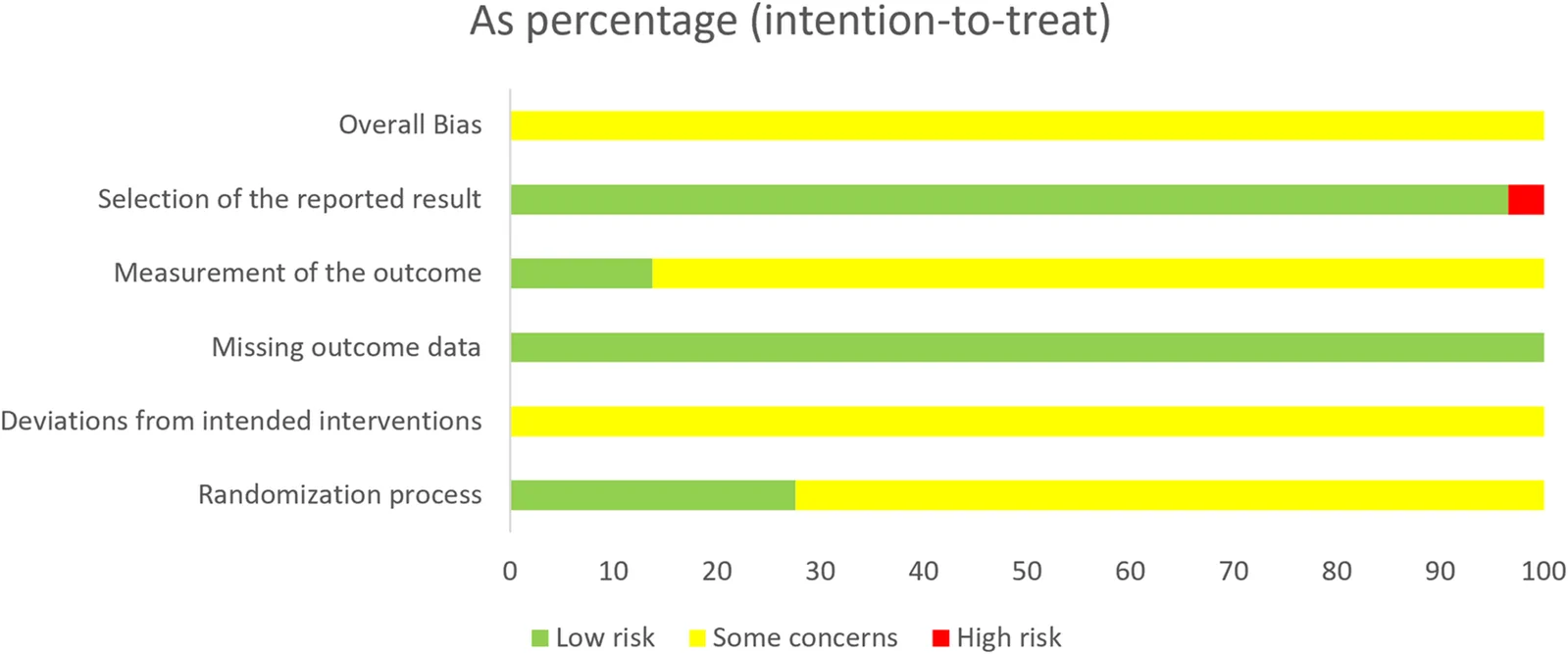
Risk of bias graph.

**Figure 3:**
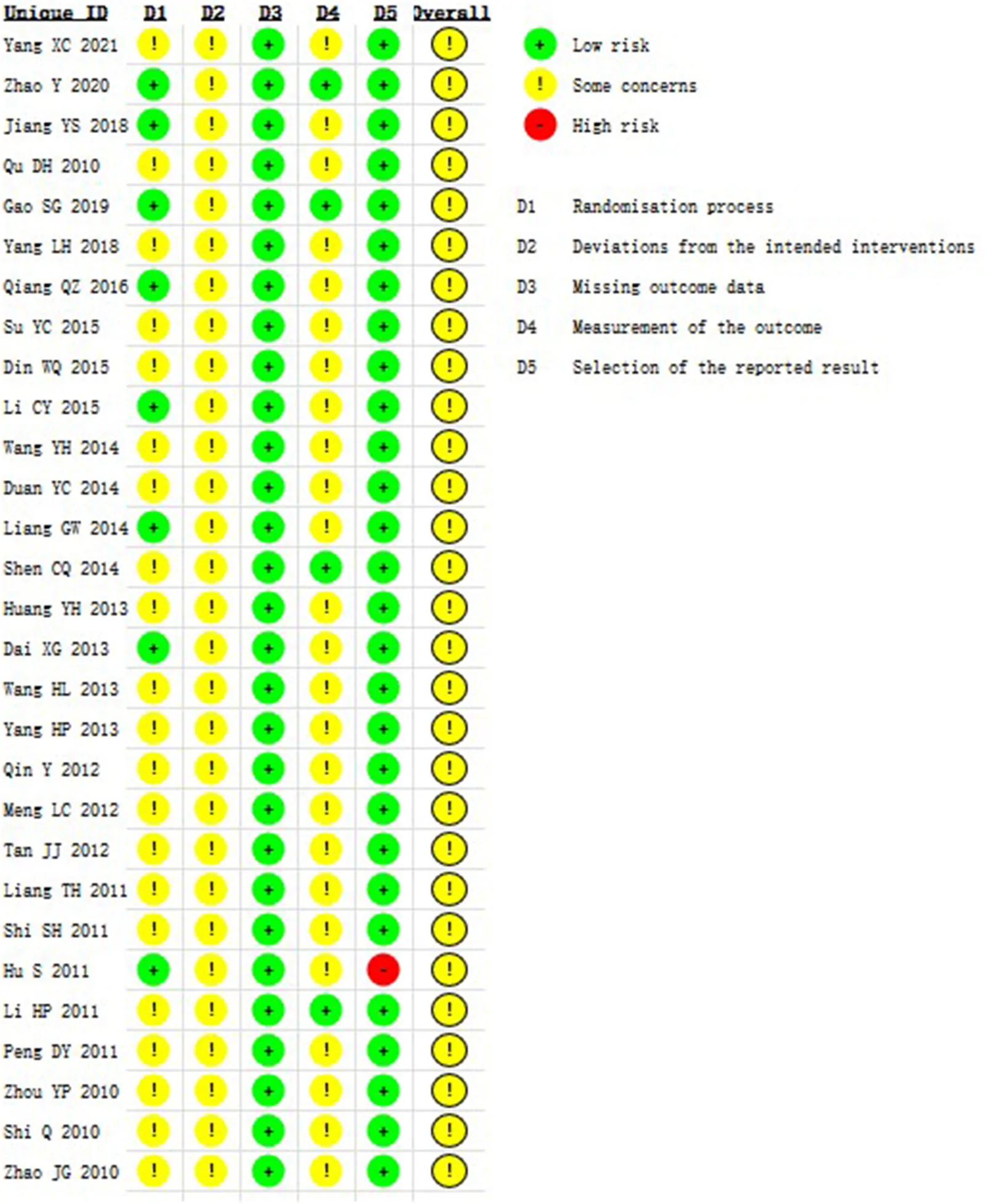
Risk of bias summary in included studies.

**Figure 4:**
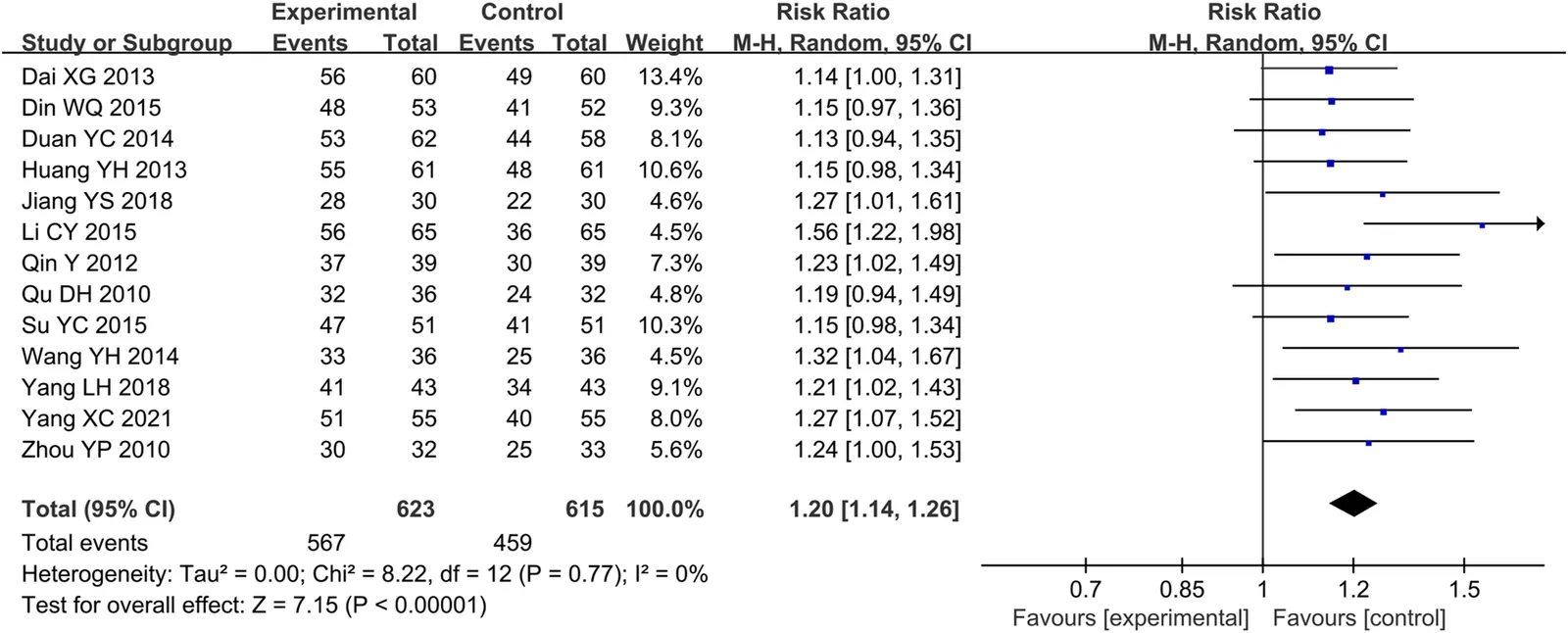
Meta-analysis of total efficacy rate. Forest plot of Breviscapine combined with conventional treatment vs. conventional treatment on total efficacy rate in CHD patients.

The evaluation of methodological quality is shown in Figures 2, 3. All 29 studies mentioned using randomization. Only 8 studies (25–27, 29, 32, 35, 38, 46) used a random number table (low risk of Randomisation process bias). The remaining 21 studies did not describe their method (some concerns). No study described hiding the treatment assignment list, so the risk of bias is rated as “some concerns” for all. No study mentioned blinding of outcome assessors. For subjective outcomes that require clinician judgment—specifically total efficacy rate and angina symptom improvement—the lack of blinding led us to rate Domain 4 (measurement of the outcome) as “some concerns” for these endpoints. Domain 4 was rated as “low risk” only for the four studies (25, 26, 36, 47). One study (46) had a high risk of selection of the reported result. For the remaining studies, the risk was low (Figures 3, 4).

### 3.4 Outcome measures

#### 3.4.1 Primary outcome measures

##### 3.4.1.1 Total efficacy rate

Thirteen studies (24, 27, 28, 30–34, 37, 38, 41, 49, 52) reported total efficacy rate, the random-effect model was used for data synthesis (meta-analysis). The *I*^2^ test showed *τ*^2^ = 0, *χ*^2^ = 8.22 df = 12, *p* = 0.77, *I*^2^ = 0%; hence, The meta-analysis showed that there was a significant beneficial effect in the experimental group compared to the control group (RR = 1.20, 95% CI 1.14–1.26; *p* < 0.00001). Based on the above results, Breviscapine demonstrates a significant therapeutic effect on the comprehensive symptoms of coronary heart disease (Figure 4).

##### 3.4.1.2 Improvement of angina symptoms

**Figure 5:**
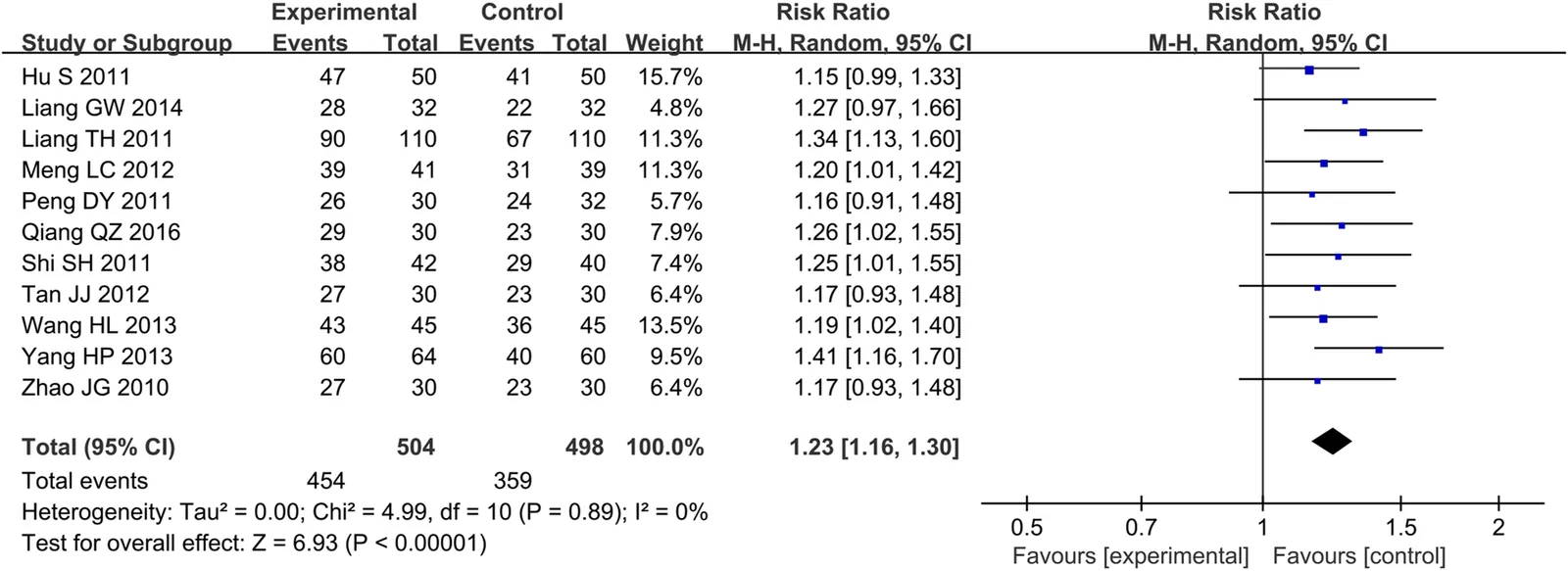
Meta-analysis of improvement of angina symptoms. Forest plot of Breviscapine combined with conventional treatment vs. conventional treatment on improvement of angina symptoms in CHD patients.

11 studies (29, 35, 39, 40, 42–46, 48, 51) reported improvement of angina symptoms and the random-effect model was used for data synthesis (meta-analysis). The *I*^2^ test showed *τ*^2^ = 0, *χ*^2^ = 4.99, df = 10 (*p* = 0.89), *I*^2^ = 0%. The meta-analysis showed that there was a significant beneficial effect in the experimental group compared to the control group (RR = 1.23, 95% CI 1.16–1.30; *p* < 0.00001). Based on the above results, Breviscapine demonstrates a significant therapeutic effect on the symptoms of angina (Figure 5).

##### 3.4.1.3 Adverse reaction

**Figure 6:**
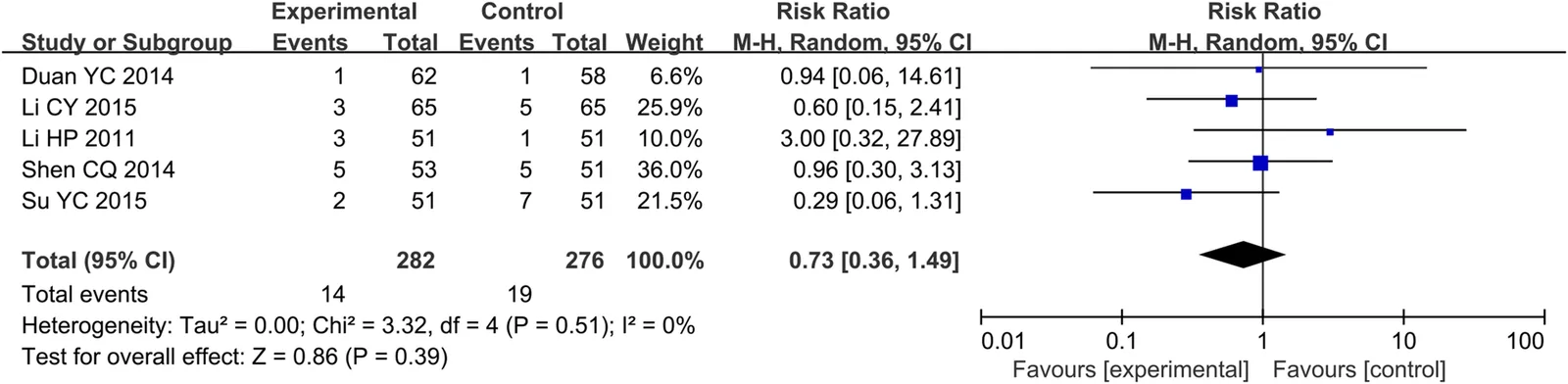
Meta-analysis of adverse reaction. Forest plot of Breviscapine combined with conventional treatment vs. conventional treatment on adverse reaction in CHD patients.

Five studies (30, 32, 34, 36, 47) reported adverse reaction. The *I*^2^ test showed *τ*^2^ = 0, *χ*^2^ = 3.32, df = 4 (*p* = 0.51), *I*^2^ = 0%. The analysis found no statistically significant difference in the number of adverse reaction between the Breviscapine group and the control group. (RR = 0.73, 95% CI 0.36–1.49; *p* = 0.39) (Figure 6).

##### 3.4.1.4 Acute cardiovascular events

Five study (39) reported that cardiovascular event rates were significantly lower in the Breviscapine group. The analysis found a statistically significant difference in the number of acute cardiovascular events between the Breviscapine group and the control group (*p* < 0.05). One study (47) reported that no acute cardiovascular events occurred. Other studies did not report this an acute cardiovascular event.

##### 3.4.1.5 Mortality

No studies reported on mortality; therefore, this outcome was not meta-analyzed.

#### 3.4.2 Secondary outcome measures

##### 3.4.2.1 Changes of electrocardiogram

**Figure 7:**
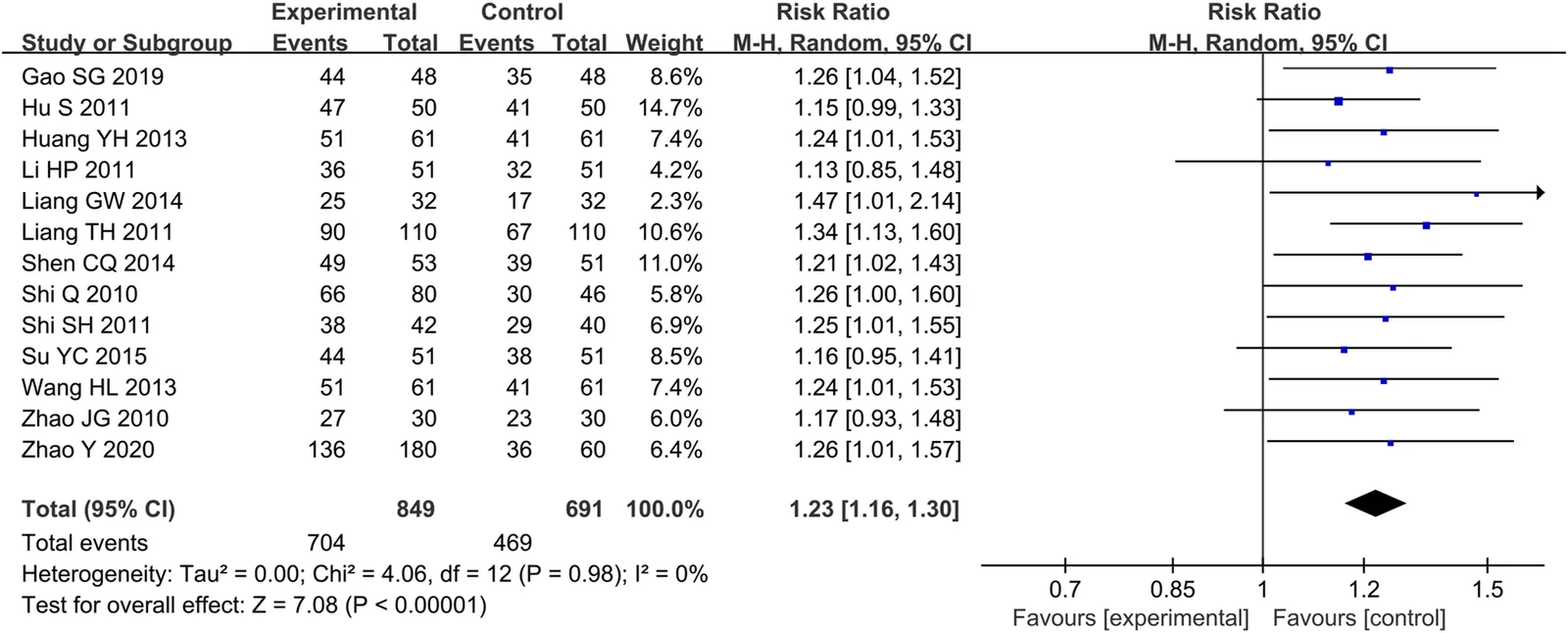
Meta-analysis of changes of electrocardiogram. Forest plot of Breviscapine combined with conventional treatment vs. conventional treatment on changes of electrocardiogram in CHD patients.

Thirteen studies (25, 26, 30, 35–37, 39, 44–47, 50, 51) reported changes of electrocardiogram and the random-effect model was used for data synthesis (meta-analysis). The analysis showed significantly greater improvement in ECG performance in the Breviscapine group (RR = 1.23, 95% CI 1.16–1.30; *p* < 0.00001) (Figure 7).

##### 3.4.2.2 Levels of lipid

Three studies (30, 32, 48) reported lipid-related outcomes. Due to substantial heterogeneity (*I*^2^ > 85% for all parameters) and the limited number of studies (2–3 per outcome), quantitative pooling was not performed. For TG, TC, and LDL-C, all three studies reported reductions in the Breviscapine group, although the magnitudes of reduction varied across studies. For HDL-C, only two studies reported this outcome; one showed an increase while the other showed no significant change. Given the substantial heterogeneity and the small number of studies, these findings should be interpreted as exploratory only.

##### 3.4.2.3 TCM syndrome score

**Figure 8:**
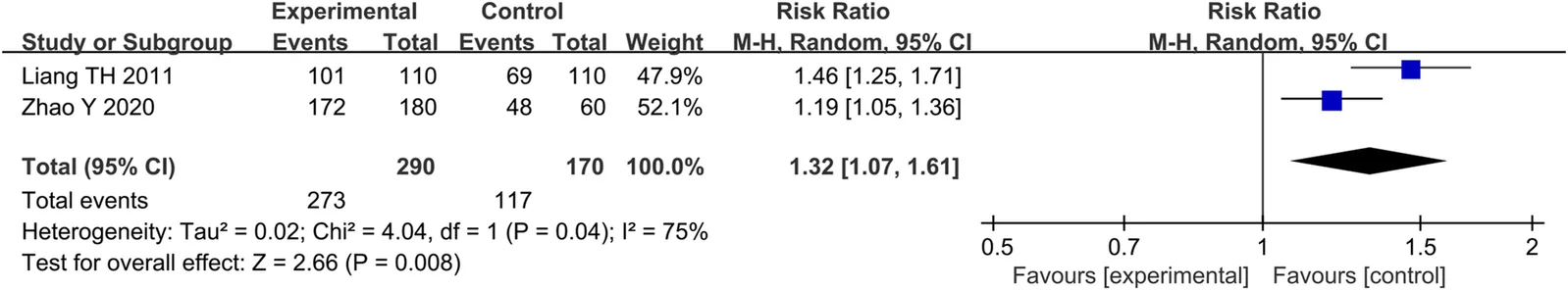
Meta-analysis of changes of TCM syndrome score. Forest plot of Breviscapine combined with conventional treatment vs. conventional treatment on changes of TCM syndrome score in CHD patients.

Two studies (25, 44) reported TCM syndrome score and the random-effect model was used for data synthesis (*τ*^2^ = 0.02, *χ*^2^ = 4.04, df = 1, *p* = 0.04, *I*^2^ = 75%). The analysis indicated that the experimental group demonstrated a more pronounced therapeutic effect in TCM syndrome compared to the control group. (RR = 1.32, 95% CI 1.07–1.61; *p* = 0.008) (Figure 8).

### 3.5 Publication bias

**Figure 9:**
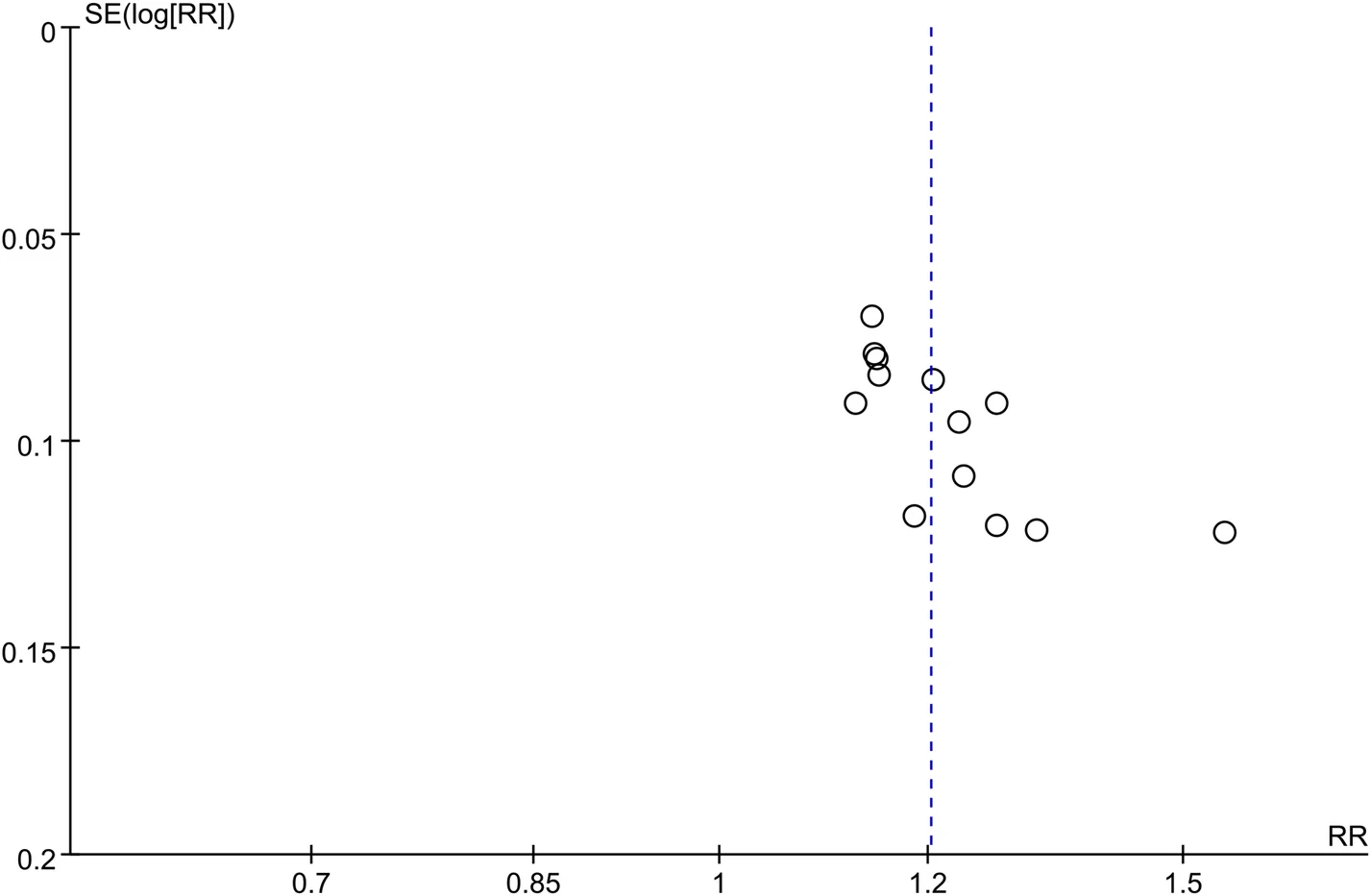
Funnel plot of total efficacy rate: Breviscapine combined with conventional treatment vs. conventional treatment on total efficacy rate in CHD patients.

**Figure 10:**
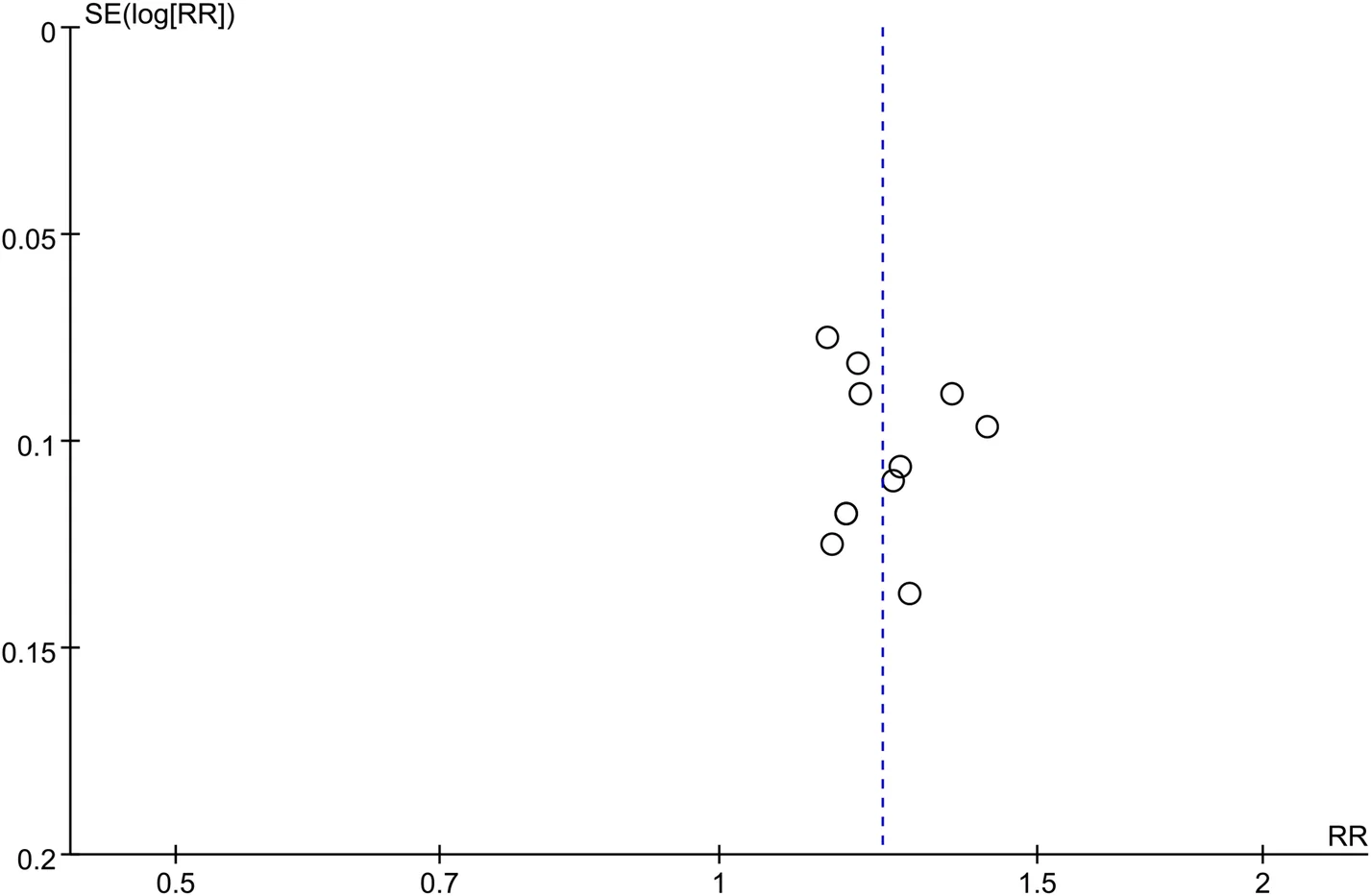
Funnel plot of improvement of angina symptoms: Breviscapine combined with conventional treatment vs. conventional treatment on total efficacy rate in CHD patients.

**Figure 11:**
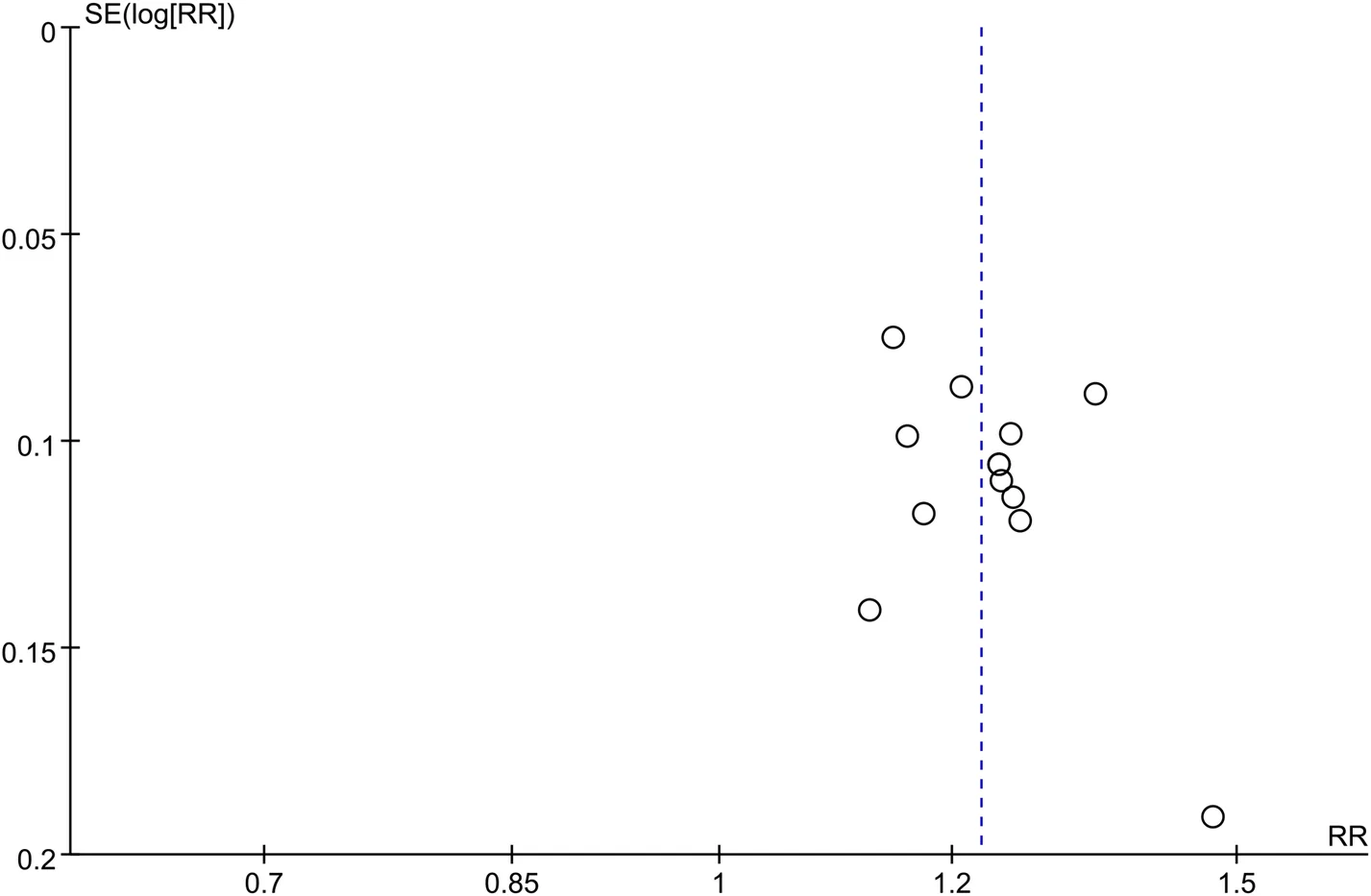
Funnel plot of improvement of changes of electrocardiogram: Breviscapine combined with conventional treatment vs. conventional treatment on changes of electrocardiogram in CHD patients.

We used funnel plots to check for publication bias for the main outcomes (total efficacy rate, angina improvement, adverse reaction). The plots showed that the points were mostly on one side but were not perfectly symmetrical, suggesting there might be some publication bias. This could be due to the small size or varying quality of the studies. All included studies were published in Chinese, which may also introduce language bias (Figures 9–11).

### 3.6 Quality of evidence

**Figure 12:**
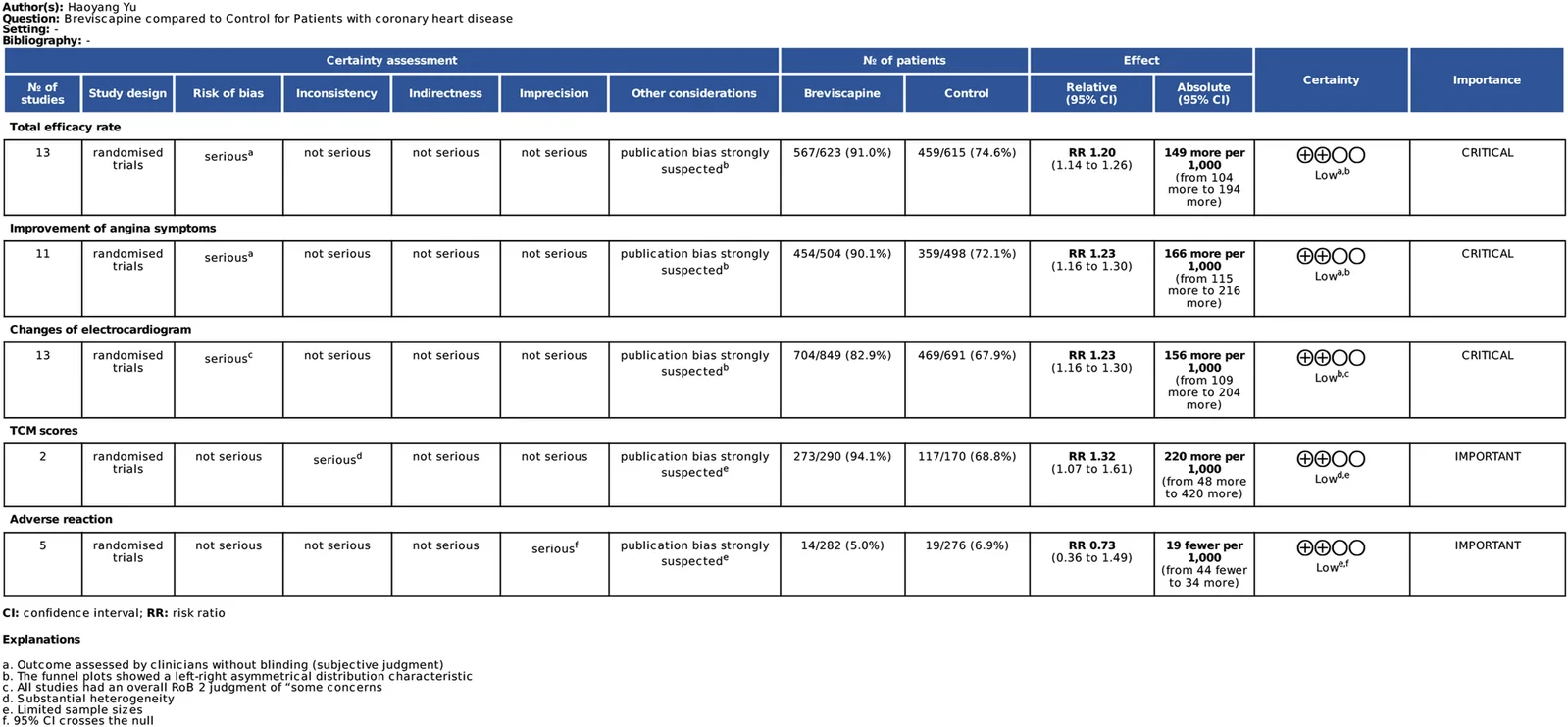
Quality of evidence assessment form (GRADE).

For total efficacy rate and angina improvement, the overall quality of evidence was rated as “low” (downgraded due to risk of bias—lack of blinding for subjective clinician-assessed outcomes—and suspected publication bias). For ECG changes, the quality was rated as “low” (downgraded for risk of bias—all studies had overall “some concerns” in the RoB 2 assessment—and suspected publication bias). For adverse reactions, the quality was rated as “low” (downgraded for imprecision due to a wide 95% CI crossing the null and suspected publication bias due to limited number of studies). For TCM syndrome scores, the quality was rated as “low” (downgraded for inconsistency due to substantial heterogeneity, suspected publication bias due to the very limited number of studies). The importance of total efficacy rate, angina improvement, and ECG changes is rated as “critical,” while the other outcomes are rated as “important” (Figure 12).

## 4 Discussion

Despite major advances in stents, surgical techniques, and modern lipid-lowering and antithrombotic drugs, coronary heart disease (CHD) still causes over 9.4 million deaths worldwide and remains the most common circulatory pathology, indicating a large residual risk and substantial burden under current care (53).

The high rate of polypharmacy is one of the major practical issues in the drug treatment of CHD. Contemporary guidelines for chronic coronary disease emphasize antiplatelet therapy, high-intensity statins, renin–angiotensin system blockers, beta-blockers, lifestyle modification, and cardiac rehabilitation, combined with percutaneous coronary intervention (PCI) or coronary artery bypass grafting (CABG) in appropriately selected patients to relieve ischemia and improve prognosis (54). Patients with CHD and other cardiovascular conditions often live with multiple chronic illnesses and therefore receive many drugs at once, which is extremely common in settings such as post-PCI, chronic coronary syndrome, heart failure, and atrial fibrillation (55). As the number of medications rises, so does the number of potential drug–drug interactions (pDDIs), including pharmacodynamic synergism, antagonism, and toxicity, and pharmacokinetic interactions via cytochrome P450, especially CYP3A4 and other isoenzymes, leading to hundreds of interaction combinations per patient cohort and markedly higher odds of adverse events and bleeding without clear additional reduction in ischemic events (55, 56). At the same time, many patients on intensive statin and standard therapy still retain substantial residual cardiovascular risk, which has driven the development and evaluation of additional lipid-modifying, anti-inflammatory, antithrombotic, and nucleic acid–based treatments that can lower events but further complicate regimens and interaction profiles (57, 58).

The outcome of surgical treatment may not be satisfactory. Large randomized trials and recent meta-analyses in stable chronic CHD with preserved left ventricular function show that an initial invasive strategy (angiography and PCI/CABG) does not reduce all-cause or cardiovascular mortality or myocardial infarction compared with optimal medical therapy, though it reduces the need for unplanned revascularization and improves angina and quality of life in symptomatic patients (59, 60).

Breviscapine is a flavonoid extract (rich in scutellarin) widely used in China. There are fundamental differences in the mechanisms of action between breviscapine and conventional Western medicines in the treatment of CHD. Conventional Western medicines (such as statins, antiplatelet agents, and beta-blockers) primarily act on single targets and single pathways, exerting their effects through lipid-lowering, antiplatelet aggregation, heart rate reduction, and other interventions. In contrast, breviscapine, as a natural flavonoid compound, exerts its cardioprotective effects through multi-target and multi-pathway synergistic regulation. Current evidence indicates that breviscapine acts through at least four major signaling pathways. First, it activates PI3K/Akt, increases eNOS and GSK-3β phosphorylation, up-regulates Bcl-2, and down-regulates Bax and caspase-3, thereby reducing cardiomyocyte apoptosis and improving function after ischemia/reperfusion and coronary microembolization (61). Second, it activates Nrf2 and downstream antioxidant proteins, decreases reactive oxygen species and oxidative damage, protecting myocardium from toxic injury (62). Third, it inhibits PKC-α-dependent ERK1/2 and PI3K/Akt cascades in hypertrophy models, reducing cardiomyocyte hypertrophy, inflammation (via NF-κB), and fibrosis (via Smad2/3) (63). Fourth, it modulates serotonin-linked PI3K/Akt signaling to increase myocardial glucose use, reduce fatty acid oxidation, enhance PINK1/Parkin-mediated mitophagy, and restore energy homeostasis, thereby alleviating cardiotoxicity and remodeling. In addition, breviscapine activates the AMPK/SIRT1/PGC1α signaling pathway, improving myocardial energy metabolism in mice while reducing lipid levels and inflammatory factors (64). This “single-compound, multi-target” mechanism allows breviscapine to simultaneously modulate energy metabolism, inflammation, apoptosis, oxidative stress, and ventricular remodeling—addressing multiple pathological processes that conventional Western medicines often target individually. This unique profile provides a mechanistic rationale for integrating breviscapine with standard therapies in CHD treatment. Therefore, rather than simply reducing polypharmacy, breviscapine should be positioned as an evidence-based add-on therapy that complements Western drug regimens via these multi-target synergistic mechanisms to address residual ischemic symptoms.

Different administration routes of breviscapine may yield varying therapeutic outcomes. Injectable (i.v.) breviscapine and its advanced injectable formulations do not change the intrinsic targets, but by rapidly achieving high systemic or brain levels, prolonging circulation, and improving tissue distribution, they may allow earlier and stronger activation of multiple pathways such as PI3K/Akt/GSK-3β, Nrf2/HO-1, and ROS/RNS–MMP-9–TJs compared with oral forms with lower bioavailability (16, 65, 66).

Integrating the above pathways, it is hypothesized that elderly patients may benefit, but this requires more studies to make confirmation. Activation of these pathways effectively dilates coronary arteries, improves microcirculation, and alleviates myocardial ischemia symptoms. Concurrently, as this population is often accompanied by chronic low-grade inflammation and oxidative stress, breviscapine can ameliorate these conditions through its multi-pathway actions. Sufficient systematic reviews are still lacking, and the data from existing reviews are rather outdated. Therefore, this study will conduct a systematic analysis of the efficacy and safety of Breviscapine in patients with coronary heart disease from the perspectives of comprehensive therapeutic effects, angina pectoris efficacy, electrocardiogram manifestations, and lipid levels, providing objective evidence.

In this study, we found that Breviscapine can effectively improve angina symptoms and electrocardiogram performance in patients with CHD, and has a certain comprehensive effect. In terms of traditional Chinese medicine symptoms (using TCM syndrome score scale), Breviscapine can also reduce the symptoms of CHD patients. At the same time, exploratory trends toward reductions in TG and LDL-C were observed across the included studies. However, due to substantial heterogeneity and limited study numbers, these findings should be interpreted with caution. Through this study, we hope to provide methods for improving symptoms, prognosis, and quality of life in patients with coronary heart disease, and to offer insights into how Breviscapine, as an evidence-based add-on therapy, can complement existing Western drug regimens via multi-target synergistic mechanisms to address residual ischemic symptoms.

Nevertheless, only three studies reported blood lipid levels, with substantial heterogeneity and limited study numbers. Therefore, we did not perform quantitative pooling for lipid outcomes; instead, findings were narratively summarized. The observed trends—all three studies consistently reported reductions in TG, TC, and LDL-C, while data on HDL-C were limited and inconclusive—should be interpreted as exploratory rather than definitive, and future studies with larger sample sizes and standardized lipid measurements are warranted to verify these observations.

In the included studies, the reporting of adverse reactions was incomplete. One study (39) reported acute cardiovascular events, and the analysis results showed a statistically significant difference between the experimental group and the control group (*p* < 0.05). Three studies (31, 39, 47) reported the effects of Breviscapine on hepatic and renal function, and all of them found no impact on liver or kidney function. However, considering the small sample sizes, there is a lack of sufficient and reliable research to fully validate the effects of Breviscapine on hepatic and renal function. The main adverse reactions in the experimental group were gastrointestinal or dermatological symptoms, which were mild and could be managed with symptomatic treatment, with some cases resolving spontaneously. However, in the current review, no precise or reliable quantitative estimate could be made regarding the possible relationship between breviscapine and adverse reactions or events. Furthermore, depending on factors such as study design and trial quality, the definitions or diagnoses of adverse reactions may vary across different trials. Further studies are needed to investigate the causal assessment of these adverse reactions. Therefore, further research is necessary to define adverse reactions to breviscapine and to investigate the factors that may contribute to its adverse reactions during long-term use.

Slight asymmetry was observed in the funnel plots for some outcomes. Given the limited number of studies and the fact that the asymmetry appeared to be driven mainly by smaller studies, formal quantitative tests (e.g., Egger's test) were not performed. Under such conditions, Egger's test may yield false-positive results and does not reliably distinguish publication bias from other sources of asymmetry. Therefore, the risk of publication bias could not be definitively judged and remains a potential limitation. In the GRADE assessment, ECG changes, total efficacy rate, angina improvement, adverse reactions, and TCM syndrome score were all rated as “low.” Among the outcomes, total efficacy rate, angina improvement, and ECG changes were rated as “critical,” while the other outcomes were rated as “important.” These findings should be considered preliminary and may serve as a basis for future high-quality studies on Breviscapine for CHD.

The overall quality of the original studies was limited. Our risk-of-bias assessment found unclear or high risk in key areas for most studies. Only 8 studies (25–27, 29, 32, 35, 38, 46) used a proper method for random assignment (like a random number table). One study (24) used an inappropriate method for random assignment; thus, the sequence generation bias was rated as high. Although all the remaining studies implemented randomized grouping, they did not employ appropriate methods such as random number tables or drawing sequences. No study described concealing the treatment allocation list from participants and personnel, which makes it difficult to judge whether the trial was conducted properly. One study (46) had a very small sample size, increasing its risk of bias.

Second, heterogeneity is another important issue that should be considered. There was significant heterogeneity in the outcomes of lipid levels and TCM score. For lipid outcomes, substantial heterogeneity and the limited number of studies precluded quantitative pooling; therefore, we summarized these findings narratively, and no further exploration of heterogeneity sources was performed. As for the TCM score, however, only two studies were included, which precluded a meaningful sensitivity analysis or subgroup analysis. By comparing these two studies in detail, we found that the heterogeneity was likely attributable to their adoption of different TCM syndrome diagnostic criteria. Due to the limited number of studies, the pooled results for TCM score should be interpreted with caution, as they may be unstable. Future meta-analyses with a larger number of high-quality studies are warranted to further validate these findings and to enable more robust exploration of heterogeneity sources.

The data on safety, especially for death or acute cardiovascular events, were very scarce—only one study reported on acute cardiovascular events (mentioned above), and none reported on mortality. The lack of long-term MACE/mortality tracking is an important limitation that should be addressed in future research. Future clinical trials on Breviscapine should be better designed. They should use methods such as proper randomization with concealment, blinding of patients and doctors, and include larger sample sizes. Although current evidence supports the use of Breviscapine for angina, larger, multicenter, high-quality randomized controlled trials are needed to make the evidence more reliable and convincing.

## 5 Conclusion

Breviscapine demonstrates good efficacy in treating angina pectoris due to coronary heart disease. It helps relieve clinical symptoms and improves electrocardiogram performance and no significant increase in short-term adverse reactions was observed. These findings support its consideration as an add-on therapy for CHD that complements, rather than replaces, conventional regimens, pending confirmation in larger-scale, high-quality trials. However, the long-term safety profile of Breviscapine remains unknown; future trials should include systematic tracking of MACE and mortality. To establish more rigorous and reliable evidence, future research should focus on conducting large-scale, multicenter, high-quality randomized controlled trials.

## Data Availability

The original contributions presented in the study are included in the article, further inquiries can be directed to the corresponding author.
